# Relaxation Time of Multipore Nanofluidic Memristors for Neuromorphic Applications

**DOI:** 10.1021/jacs.5c04903

**Published:** 2025-05-11

**Authors:** Gonzalo Rivera-Sierra, Patricio Ramirez, Juan Bisquert, Agustín Bou

**Affiliations:** 1 Instituto de Tecnología Química (Universitat Politècnica de València-Consejo Superior de Investigaciones Científicas), Av. dels Tarongers, València 46022, Spain; 2 Dept. de Física Aplicada, Universitat Politècnica de València, València E-46022, Spain; 3 Leibniz-Institute for Solid State and Materials Research Dresden, Helmholtzstraße 20, Dresden 01069, Germany

## Abstract

Memristors have been positioned at the forefront of the purposes for carrying out neuromorphic computation. Their tunable conductance properties enable the imitation of synaptic behavior. Nanofluidic memristors made of multipore membranes have shown their memristic properties and are candidate devices for liquid neuromorphic systems. Such properties are visible through an inductive hysteresis in the current–voltage sweeps, which is then confirmed by the inductive characteristics in impedance spectroscopy measurements. The dynamic behavior of memristors is largely determined by a voltage-dependent relaxation time. Here, we obtain the kinetic relaxation time of a multipore nanofluidic memristor via its impedance spectra, modeling it and deriving a general equation for this time as a function of the applied voltage, fully correlated with the system’s internal parameters. We show that the behavior of this characteristic of memristors is comparable to that of natural neural systems. Hence, we open a way to study the mimic of neuron characteristics by searching for memristors with the same kinetic times.

## Graphical abstract

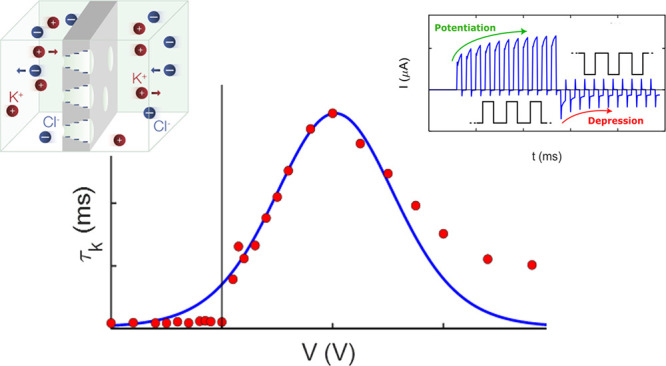

## 1 Introduction

The rapid development of emerging memory technologies has fueled the search for novel materials and device architectures to overcome the limitations of traditional memory technologies.^1^ Memristors, as a promising candidate in this field, are set to play a crucial role.^2,3^ Their unique ability to retain a history of past voltages and currents, effectively functioning as a memory, offers a significant advantage in terms of power efficiency and speed.^4^ These devices can be integrated into neuromorphic systems, which mimic the neural architectures of the human brain, paving the way for more advanced and efficient artificial intelligence applications.^5–7^ With a growing demand of processing power due to the growth of such applications, the candidates for realizing memristic devices is increasing.^8,9^

Understanding the operation and performance of memristors is of crucial importance to implement such devices in real neuromorphic systems. However, given the variety of different material and architecture candidates for building memristic functional devices,^10–13^ it is advantageous to have specific characteristics that permit inspecting the suitability of memristors for mimicking synaptic and neural behavior.^14^ Among these characteristics, we focus our research on their kinetic relaxation time. This element governs the speed of the transitions from one conductive state to another, and it is a crucial element of neuron models such as the Hodgkin-Huxley model.^15^ The velocity of these transitions depends on the applied voltage and so it does the kinetic time. The analysis of this characteristic of memristors is of use when finding similarities with relaxation times of neuron models. When a memristor has a relaxation time with the adequate voltage dependence, it is suitable for its integration in neuromorphic systems. In addition, the relaxation determines the characteristics of switching times that usually present exponential dependence with the voltage in solid state memristors that contain ionic and electrochemical processes.^16–20^

The iontronic nanofluidic channels^21–24^ have shown the characteristic inductive hysteresis of memristors, as well as the gradual tunable conductive potentiation desired for neuromorphic applications.^25–30^ However, the relaxation time of these devices has not been yet explored. Here, we propose Impedance Spectroscopy (IS) as a potential tool for accessing this characteristic of memristors. Our approach suggests to look at the IS produced by neuronal systems and synapses exploiting the similarities in the IS experiments of memristors.^31^ The key element for a neuromorphic applications of a memristor is the inductive low frequency arc,^32^ and here we obtain the associated kinetic time.

Interestingly, there have been various reports using this technique, which have found similarities in the experimental IS spectra of memristors with those given by various neuron models. Specifically, the nanofluidic memristors made of multipore membranes have already been reported to show IS spectra with sharp inductive elements.^32^ Halide perovskite memristors have shown this type of inductive spectra, too, and they have been used to correlate the IS inductive behavior with the hysteretic current–voltage curves characteristic of memristors.^33,34^

In a recent model, we have shown that both the inductive and the capacitive conduction effects can be described with a single relaxation equation producing both phenomena.^35^ Therefore, we can conclude that both inductive and capacitive traces have the same physical origin. In fact, we simplify the equivalent circuit used for the IS analysis, and we can describe the full set of spectra with a single R-L branch.

This approach allows us to extract new information about the characteristics of memristors. Here, we apply our model to the analysis of multipore nanofluidic memristors with synaptic tunability.^27^ We fit the experimental IS spectra of the memristor and analyze the behavior of various parameters as a function of the applied voltage, including the relaxation time of the system that governs the conductance change in the memristor. These behaviors align with theoretical predictions derived from the application of a small-signal perturbation to our equations. In addition, in this work, we establish an exact functional dependence of the relaxation time on the applied voltage, relying solely on the system intrinsic and geometric parameters. With this approach, we achieve full simulation and control of neuromorphic applications enabled by these memristors, thereby laying the foundation for the effective correlation and integration of memristors with neuromorphic systems.

## 2 Model

The properties of the pore and methods of measurements of *I*–*V* curves are described in Supporting Information. The membrane contains approximately 300 asymmetric nanopores with conical geometry, around 12.5 μm in length, and diameters on the order of 200 nm at the base and 20 nm at the tip, as determined from current–voltage curve fitting. A schematic representation of the setup is shown in Figure 1.

**Figure 1:**
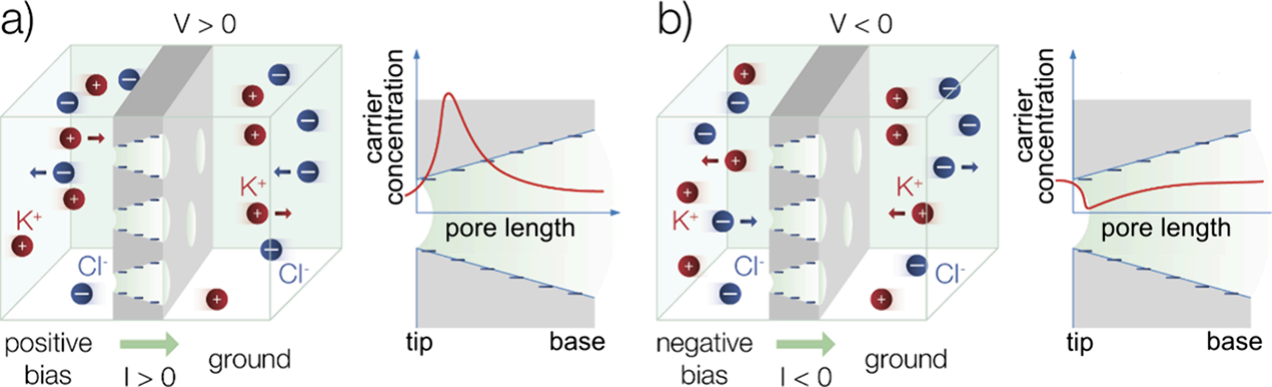
Schematic representation of the mechanism of rectification that is obtained when the voltage applied to the cell is inverted in polarity giving higher (a) or lower (b) current according to the mobile charge carriers accumulation or depletion in the tip of the pore with negatively charged walls.^39^ **Reproduced from** Bisquert, J.; Sanchez-Mateu, M.; Bou, A.; Suwen Law, C.; Santos, A. *Synaptic Response of Fluidic Nanopores: The Connection of Potentiation with Hysteresis*, *ChemPhysChem* **2024**, 25, e202400265. Licensed under a Creative Commons Attribution (CC BY 4.0) license.

### 2.1 Relation of Conductance to Pore Charges

Starting from the fundamental basis, the stationary and dynamic currents in our membranes are intrinsically determined by the geometry of the pores.^23,36^ Typically, these pores are conical in shape, resulting in a high negative fixed charge density at the pore tip, which progressively decreases toward the pore base. As a consequence, the profiles of electric potential and mobile charge carriers become highly nonlinear in the region of the pore tip, where the effects of fixed charges are more pronounced.^37,38^ At *V* > 0 (Figure 1a), the electric field pulls the counterions toward the pore tip, where they accumulate, leading to a maximum in the concentration profile. The co-ions are also driven to the pore tip to maintain electroneutrality, and the corresponding concentration profile resembles that of the counterions. Consequently, the total concentration of charge carriers at the pore tip increases with the applied voltage, and hence *I* increases rapidly with V. For V < 0 (Figure 1b), the electric field drives the counterions out of the pore tip, the co-ions follow the same trend in order to preserve electroneutrality, and the profile of total concentration of charge carriers attains a minimum. The depleted concentrations of charge carriers give now significant lower conductance than in the case V > 0, and a quasi-linear increase of I with V occurs.^37,38^ This asymmetric response of the mobile charge carriers to the polarity of the applied voltage results in the current rectification shown in Figure 2.

**Figure 2:**
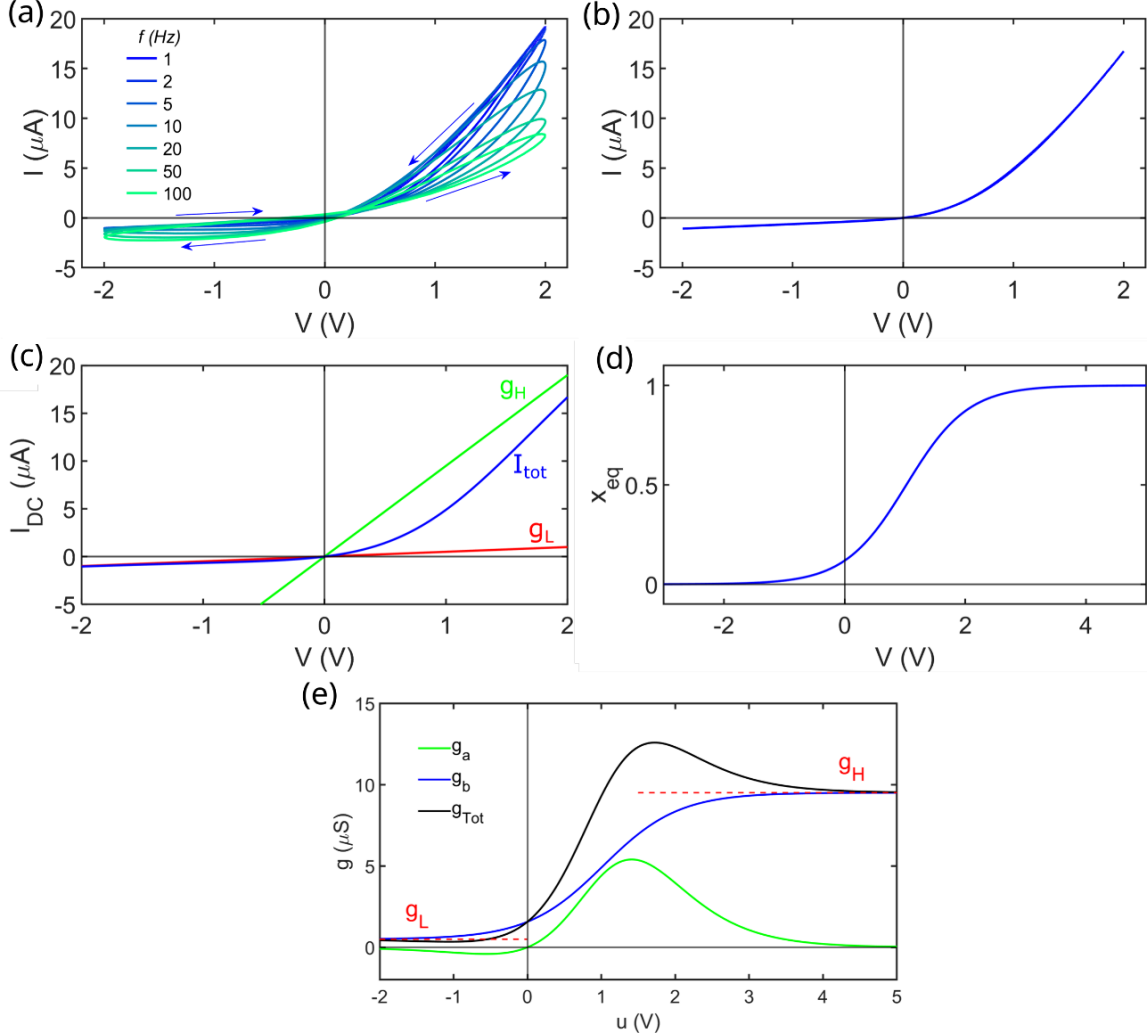
Experimental (a) Current–voltage curve of the device applying a triangular 2 V amplitude voltage signal with a sweep rate of 200 *mV*/*s* and (b) applying a 2 V amplitude sinusoidal voltage signal *V* = *U*_0_ sin (2π*ft*) at different cycle frequency values *f*. Simulated (c) current–voltage curve of the device in blue, additionally showing the low conductance branch in red and the high conductance branch in green, and (d) system memory variable in the stationary as a function of voltage, and (e) Dynamic model representation of the conductance values of each branch and the total conductance (*g*_*tot*_ = *g*_*a*_ + *g*_*b*_), the model equations are described by (10–11). The parameters used for the simulations are those obtained from fitting the experimental current–voltage curve of (a).

When the frequency of the voltage signal is relatively low (Figure 2a), the I–V curve shows no hysteresis because the signal period is longer than the relaxation time, which characterizes the interaction between the mobile carriers and the fixed pore charges. When the frequency is increased to the 1–100 Hz range (Figure 2b), significant hysteresis loops appear because the signal period becomes shorter than this relaxation time, and memristive behavior arises.^23^

The profiles outlined in Figure 1 indicate that the total concentration of mobile carriers at the nanopore tip changes drastically compared to the background concentration along the nanopore as we vary the voltage. We denote the concentration of mobile carriers at the pore tip as *X*, which increases for positive voltages and decreases for negative voltages relative to the standard background concentration *c*, and define a normalized state variable *x* related to the concentration of carriers at the pore tip and its corresponding maximum (*X*_*ac*_) and minimum (*X*_*dep*_) values as

$$x=\frac{X-X_{dep}}{X_{ac}-X_{dep}}$$ 1

In this way, *X* reaches a constant maximum value *X*_*ac*_ at large positive voltages and a constant minimum value *X*_*dep*_ at large negative voltages. Correspondingly the state variable *x* changes between 1 and 0. The existence of these limiting values is supported by the fact that conductance remains constant at high enough positive and negative voltages, with further current variation being solely due to changes in voltage and no further changes in *X* occurring.

Now, based on this predefined state variable derived from the chemical-physical behavior of our device and relying on the previously studied theoretical current–voltage model exhibited by this type of systems,^35,39^ we derive the following dynamic equations for the system

$$I_{tot}\left(u\right)=\left[g_{L}+\left(g_{H}-g_{L}\right)x\right]u$$ 2

$$\tau_{k}\left(u\right)\frac{dx}{dt}=x_{eq}\left(u\right)-x$$ 3

The system current *I*_*tot*_ is described by eq 2 and varies between two conductance states, high (*g*_*H*_) and low (*g*_*L*_), through changes in the normalized system memory variable *x*. This variable evolves according to the dynamics of eq 3, where *x*_*eq*_ represents its voltage-dependent equilibrium value in the steady-state regime, and τ_*k*_ is the relaxation time associated with the change in this variable and, thus, the entire system. Since τ_*k*_ is voltage-dependent, it naturally introduces dynamic asymmetry and rate sensitivity into the system’s response, which is reflected in the shape and extent of the hysteresis observed in *I–V* curves under varying signal conditions.

In the stationary states, the model takes the following form

$$x_{eq}\left(u\right)=\frac{1}{1+e^{-\left(u-V_{Bx}\right)/V_{m}}}$$ 4

$$I_{DC}\left(u\right)=\left[g_{L}+\left(g_{H}-g_{L}\right)x_{eq}\right]u$$ 5

where the equilibrium form *x*_*eq*_ is a typical sigmoidal function with onset parameter *V*_*Bx*_ and steepness *V*_*m*_. Thus, the stationary parameters governing our system can be directly obtained from a fitting of the experimental stationary current–voltage curve, Figure 2a. In Figure 2(c-e) we show the stationary parameters that rule our experimental system, and we can notice that the variable *x*_*eq*_ gradually drives the system from the low-conductance state at large negative voltage values (scenario (b) at Figure 1) to the high- conductance state at large positive voltage values (scenario (a) at Figure 1).

### 2.2 Dynamic Behavior

At this point, we have described and modeled the system steady-state behavior. However, we cannot directly scale to describing its dynamics. To deeply analyze the properties of this behavior, we calculate the small signal ac impedance response at the angular frequency ω. As usual the equations are expanded to the first order,^40,41^ where the perturbation of variable *y* is indicated as *ŷ*, and the factor functions of each term are computed at equilibrium conditions. Furthermore, we transform the small signal equations to the frequency domain by the Laplace transform, *d*/*dt* → *s*, where *s* = iω. We obtain the equations^35^

$${Î}_{tot}=\left[g_{L}+\left(g_{H}-g_{L}\right)x_{eq}\left(u\right)\right]û+\left(g_{H}-g_{L}\right)u\mathrm{x̂}$$ 6

$$\mathrm{x̂}=\frac{c_{\mu }}{1+s\tau_{k}}û$$ 7

Here

$$c_{\mu }=\frac{dx_{eq}}{du}$$ 8

plays the role of a chemical capacitance (here with dimension *V* ^–1^).^42^ The solution of the impedance obtained from (6–8) is

$$Z\left(s\right)=\frac{û}{{ĵ}_{tot}}={\left[g_{b}+\frac{g_{a}}{1+s\tau_{k}}\right]}^{-1}$$ 9

The circuit elements are defined by the relationships

$$g_{b}=g_{L}+\left(g_{H}-g_{L}\right)x_{eq}\left(u\right)$$ 10

$$g_{a}=c_{\mu }\left(u\right)\left(g_{H}-g_{L}\right)u$$ 11

$$L_{a}=\frac{\tau_{k}}{g_{a}}$$ 12

Here *g*_*a*_ represents the low conductance of the equivalent circuit model, dynamically controlled by the inductor *L*_*a*_ in series with the resistor, and *g*_*b*_ corresponds to the high conductance, acting in parallel with the *L*_*a*_ and *g*_*a*_ branch. The inductor element corresponds to a chemical inductor^43^ that plays a dominant role on inverted hysteresis^44^ and synaptic potentiation.^33,45^

The voltage-dependent behavior of the system conductances can be derived from the parameters and equations obtained in the static regime, *g*_*H*_, *g*_*L*_, *x*_*eq*_ and *c*_μ_.

Figure 2e presents the system conductances extracted from the development of the model dynamics, including the total conductances calculated from the sum of both conductances. The conductance *g*_*b*_ shows a gradual change from the low conductive state to the high conductive state like that of the variable *x*_*eq*_. We highlight the behavior of *g*_*a*_ which takes positive and negative values. Here, we observe the negative values at negative voltages, i.e., at the low conductive state, and positive values at positive voltages, i.e. at the high conductive state. However, a complete description of the system kinetics also requires obtaining the relaxation time function τ_*k*_(*u*).

### 2.3 Relaxation Time Model

To accurately describe the system relaxation time with a sophisticated and coherent model that could align with experimental results, we must first understand the fundamental principles and complete functioning of the system from the ground up.

Considering the model and the previously described eqs 1–3, which relate the chemical-physical behavior of the experimental system to the models that precisely describe its electrical response, and in order to obtain a model for the relaxation time as a function of the voltage applied to the system, we extend the discussion by Mafe et al.^46^ Under a voltage step, the current flow required to change the charge density at the pore tip is determined by the following conservation equation

$$q\frac{\partial X}{\partial t}=-\frac{\partial J_{ch}}{\partial y}$$ 13

where *J*_*ch*_ is the drift current density and can be described as

$$J_{ch}=qc\mu_{X}E_{ch}$$ 14

Here μ_*x*_ is the carrier mobility, which is related to the diffusion coefficient *D*_*x*_ through the following relationship

$$\mu_{X}=\frac{qD_{X}}{k_{B}T}$$ 15

where *k*_*B*_ *T*/*q* is the product of the Boltzmann constant and the absolute temperature divided by the elementary charge, also known as the thermal voltage *V*_*T*_.

Under a positive voltage step, the electric field in the channel, described in eq 14, would always be negative, as it increases the charge density at the pore tip. Thus, we can write

$$E_{ch}=\frac{\Delta V}{L}$$ 16

where *L* is the pore length, and Δ*V* is the voltage difference applied to the system. Starting from the continuity equation we can express

dX=ΔX·dx 17

where Δ*X* is the constant difference *X*_*ac*_ – *X*_*dep*_. From

$$\frac{\partial J_{ch}}{\partial y}=\frac{-qc\mu_{x}\Delta V}{L^{2}}$$ 18

we arrive at

$$\Delta X\frac{dx}{dt}=\frac{c\mu_{x}\Delta V}{L^{2}}$$ 19

Introducing this expression for the derivative *dx*/*dt* into the dynamic equation for *x* (3), and relating (*x*_*eq*_ – *x*) to the first term of the Taylor series that describes the voltage change respect to the change in the state variable *x*

$$\Delta V=\left(\frac{dV}{dx}\right)\left(x_{eq}-x\right)$$ 20

we obtain

$$\tau_{k}=\frac{\Delta XV_{T}L^{2}}{cD_{x}}\frac{dx}{dV}$$ 21

Defining the diffusion time as τ_*D*_ = *L* ^2^/*D*_*x*_, we arrive at the final equation that would govern the behavior of the system relaxation time at different voltage values

$$\tau_{k}=\gamma_{0}V_{T}^{\alpha }{\left(\frac{dx}{du}\right)}^{\alpha }+t_{0}$$ 22

where

$$\gamma_{0}=\tau_{D}\frac{\Delta X}{c}$$ 23

We have introduced a voltage modulation parameter α to appropriately modulate and adjust the relaxation time obtained at different voltages under any experimental condition, as well as a background time *t*_0_ associated with the system intrinsic experimental response time. Additionally, we also have approximated the applied voltage *V* with the system internal voltage *u* (*V* ≈ *u*), thereby neglecting the resistance potentially present in the electrodes and solution.

The relaxation time of eq 22 can also be expressed in a more intuitive way. By defining the nanopore resistance as *R*_*p*_ = *V*/(*J*_*ch*_ · *A*), the relaxation time can be witten as

$$\tau_{k}=q\Delta XR_{p}AL\frac{dx}{du}$$ 24

Here *A* is the cross-sectional area of the pore tip, and although Δ*X* has been described as a constant, it has dimensions of carriers concentration. Thus, multiplying this constant by the pore volume, we can obtain the difference in the number of accumulated carriers in the pore between its maximum and minimum values, i.e between the two conductive states of the system. By further multiplying this value by the elementary charge, we can determine the total effective charge *Q* = *q*Δ*XAL* that contributes to the formation of the chemical capacitance *C*_μ_. In this way, we obtain *C*_μ_ = *Qc*_μ_, being *c*_μ_ the chemical capacitance of the pore with *V* ^–1^ dimensions in eq 8 and *C*_μ_ with real capacitance dimensions. Thus, we arrive to the universal form of the relaxation time of an ionic charging system

$$\tau_{k}=R_{p}C_{\mu }$$ 25

To apply this model and ensure it fully aligns the experimental results, we introduce the parameters α and *t*_0_ again, leading to

$$\tau_{k}=R_{p}V_{T}^{\alpha -1}Qc_{\mu }^{\alpha }+t_{0}$$ 26

Equations 22 and 26 are main results of this work. We have commented before that a main property of memristors described by eqs 2 and 3 is the transition of conductance governed by the variable 0 ≤ *x* ≤ 1. But another important piece of the model is the relaxation time τ_*k*_ that determines the kinetics under time-varying perturbation. Furthermore, this time should be valid over the whole voltage domain where the transition of conductance (i.e., the change of *x* from 0 → 1) occurs. In this way, the dependence of the internal variable *x* on voltage will directly influence the shape of the relaxation time. The variation of the internal variable *x* as a function of its parameters is shown in Figure 3a, while the resulting relaxation time, derived from eq 22, is depicted in Figure 3b. It provides a peaked-shaped relaxation time, reminiscent of the Hodgkin-Huxley relaxation times for the protein channels in the cell membrane.^15,47,48^ Clearly, the relaxation time shape dependence on voltage follows the derivative in eq 8, while the time scale is set by the parameter γ_0_.

**Figure 3:**
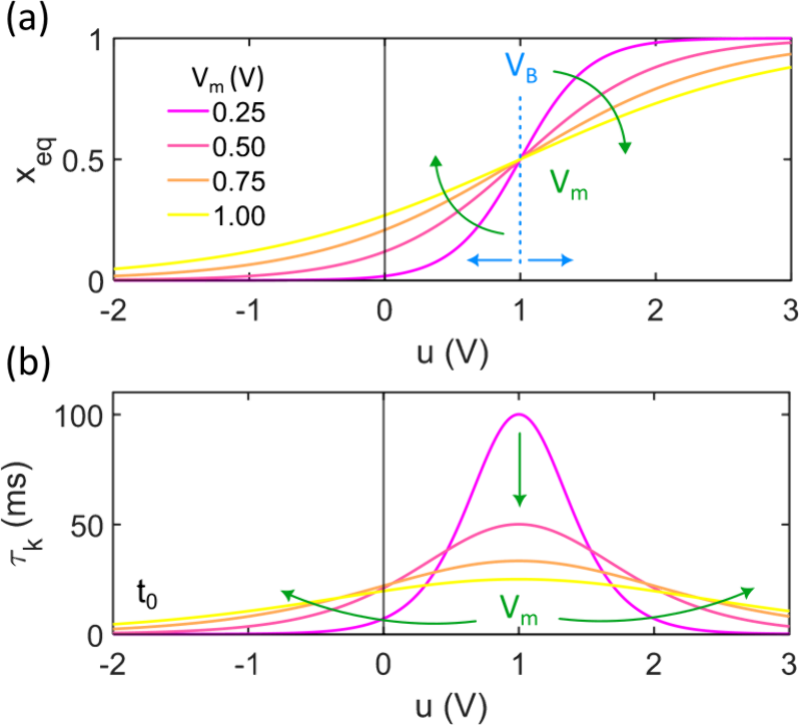
Simulation of (a) the internal variable *x* as a function of voltage for different values of the parameters that determine it, and (b) the resulting relaxation time obtained through eq 22. The model in (a) is described based on eq 4, whereas the model in (b) is derived from the fitting equation τ_*k*_(*u*) = *Kc*_μ_^α^(*u*) + *t*_0_, where *K* is an adjustable parameter that can be defined to obtain different internal parameters based on both eqs 22 and 26. The parameter values used for all simulations are *V*_*B*_ = 1.00 *V*; *A* = 0.10 *sV* ^α^; α = 1; *t*_0_ = 0.10 *ms*.

## 3 Results

The methods of measurement of ac Impedance have been described in the Supporting Information.

### 3.1 Impedance Spectroscopy

To validate the model and obtain significant information toward the neuromorphic behavior, we perform impedance measurements on our system at different voltage values. This approach will allow us to extract intrinsic system parameters, such as the conductances of the various branches, the inductor values and the system relaxation time. Recent papers have explained the general structure of the IS of memristors, in relation to the equivalent circuit that contains resistors, capacitors and inductors.^35,45^

In Figure 4 we represent the experimental impedance spectrum measured in our system and fit them to the model that describes it. We show in Figure 4a the experimental IS data, where we can observe the previously reported transition^32^ from a two capacitive arcs spectrum at low conductive state or negative voltages, to an inductive low frequency arc spectrum at high conductive state or positive voltages. This form is analogously observed in halide perovskite memristors.^33^ Additionally, the onset of a small capacitive arc at very low frequency can be observed, which becomes more evident at higher voltage values and is associated with the accumulation of ionic charge on the pore walls at high voltages and over long time scales.^49^

**Figure 4:**
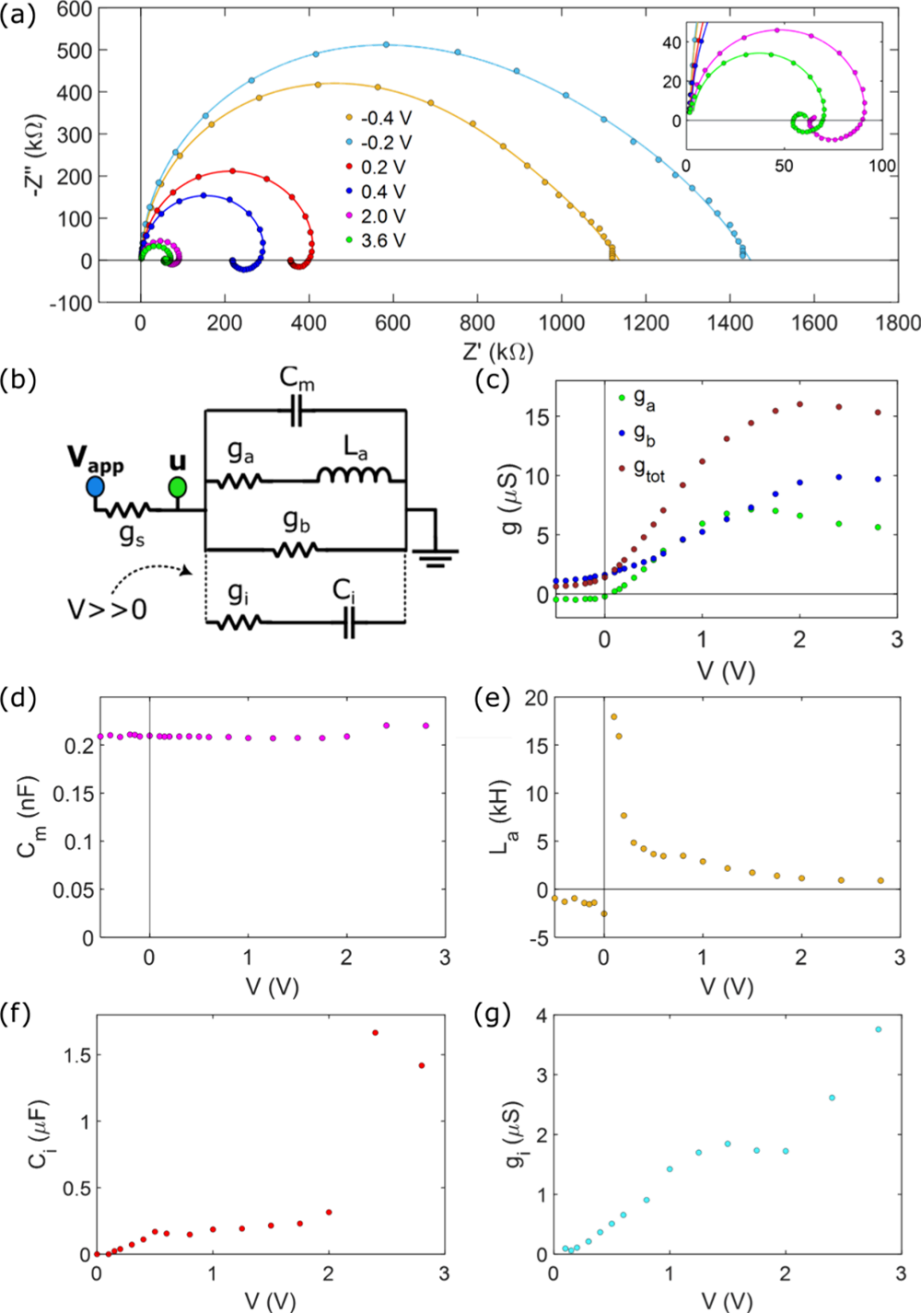
IS experimental data and analysis. (a) IS spectra at different voltages, points are the experimental data and straight lines are the fitting results. (b) Equivalent circuit used for the data fitting. Extracted parameters: (c) Conductance. (d) Membrane capacitance. (e) Inductance. (f) Capacitance and (g) conductance of the ionic accumulation branch.

The equivalent circuit is shown in Figure 4b, where the (*g*_*a*_. *g*_*b*_, *L*_*a*_) subcircuit corresponds to the model of eq 9, where we have added an ionic RC branch, in order to fit this small capacitive arc at large positive voltages,^49^ and a high frequency capacitor, in order to fit the high frequency arc present in all the membrane devices.

In the remaining graphs of Figure 4 we present the behavior of the equivalent circuit elements extracted from the impedance spectra fitting.

Figure 4c shows the conductances of the different branches of the system. *g*_*b*_ takes only positive values transitioning from a low conductance to high conductance, *g*_*a*_ changes from negative values to positive values at the transition region from low to high conductive state. As seen in Figure 2e, *g*_*tot*_ reaches its maximum at a certain voltage after the transition and then decays slightly, attempting to reach the plateau.

In Figure 4d, we observe how the capacitor connected in parallel with the entire system, which is common in all membrane devices of this type, remains constant across all voltage values, as expected. This behavior is inherent to the membrane and is not influenced by the external excitation. The inductor is shown in Figure 4e. This parameter can be related to the conductance of its corresponding branch through eq 12, allowing us to obtain relaxation time values for the experimental system and, thus, the experimental overall dynamics of our device. These values will be presented alongside their fit to the previously described model in the following section. The final parameters displayed in Figures 4f and 6g represent those replicating the ionic charge accumulation properties within the pore.

### 3.2 Determination of the Relaxation Time

By following the experimental relaxation time obtained from the parameters extracted from the IS measurements in our device and fitting these data points to the previously described model in eqs 22 and 26, we can initially assess the feasibility of the model and determine the exact values that our system provides to describe it. The results are shown in Figure 5. We remark that the model agrees very well with the measurements, although at high positive voltage another ionic accumulation effect creates a different dependence.

**Figure 5:**
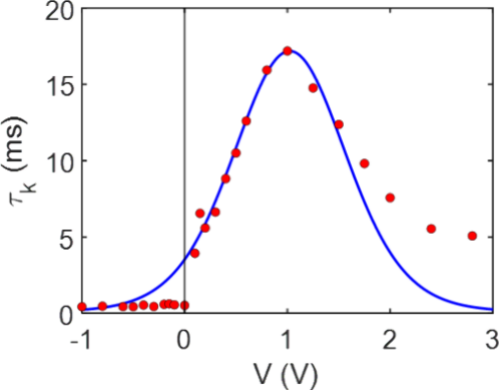
Analysis of the relaxation time. Experimental data (dots) obtained by eq 12 and fit to the model τ_*k*_ = *Kc*_μ_^α^ + *t*_0_ (line), previously used in Figure 3b. Adjusted parameters: *K* = 0.064 *sV* ^α^ ; *V*_*B*_ = 1.023 *V* ; *V*_*m*_ = 0.511 *V* ; α = 1.849 ; *t*_0_ = 0.104 *ms*.

The fitting parameters are shown in the figure caption, and by applying these fitting parameters to both eqs 22 and 26, we can obtain values for the system parameters, such as γ_0_ = 54.6 *s* and *R*_*p*_ *Q* = 1.42 Ω*C*. This is commented further in the Discussion section.

### 3.3 Neuromorphic Performance

To evaluate and neuromorphically apply these fluidic memristors, we conducted typical synaptic measurements. It is well established that such systems exhibit synaptic behavior,^27–29,39^ but these behaviors have not been well replicated or controlled based on the direct theoretical parameters of the system.^23^ Subsequently, these experimental measurements will be fully replicated theoretically through simulations of the complete model, incorporating the response time, thereby confirming the validity of the relaxation time model and its control over neuromorphic applications solely through the parameters and properties of the system comprehensive model.

We begin by presenting the experimental response of the system under different voltage pulse configuration. First, in Figure 6a, the system response to a typical applied voltage pulse of 1.25 *V* is shown. Here, we observe an exponential increase in current until reaching the stationary current value at this voltage. This current response to a voltage pulse in such systems is neuromorphically referred to as excitatory postsynaptic current (EPCS), which plays crucial role in all these applications. Following this, Figure 6b illustrates the well-known synaptic potentiation-depression behavior.^27,50,51^ Initially, a train of short positive pulses, in this case 11 pulses of 2 *V*, is applied, followed by the same train of pulses but in the negative polarity, i.e – 2 *V*. With the positive pulses, the current increases incrementally with each pulse, attempting to reach a stationary value. Conversely, with the negative ones, the same phenomenon occurs but with a decrement in current. This modulation of the internal memory of current when short pulse trains are applied is referred to as potentiation and depression, respectively.

**Figure 6:**
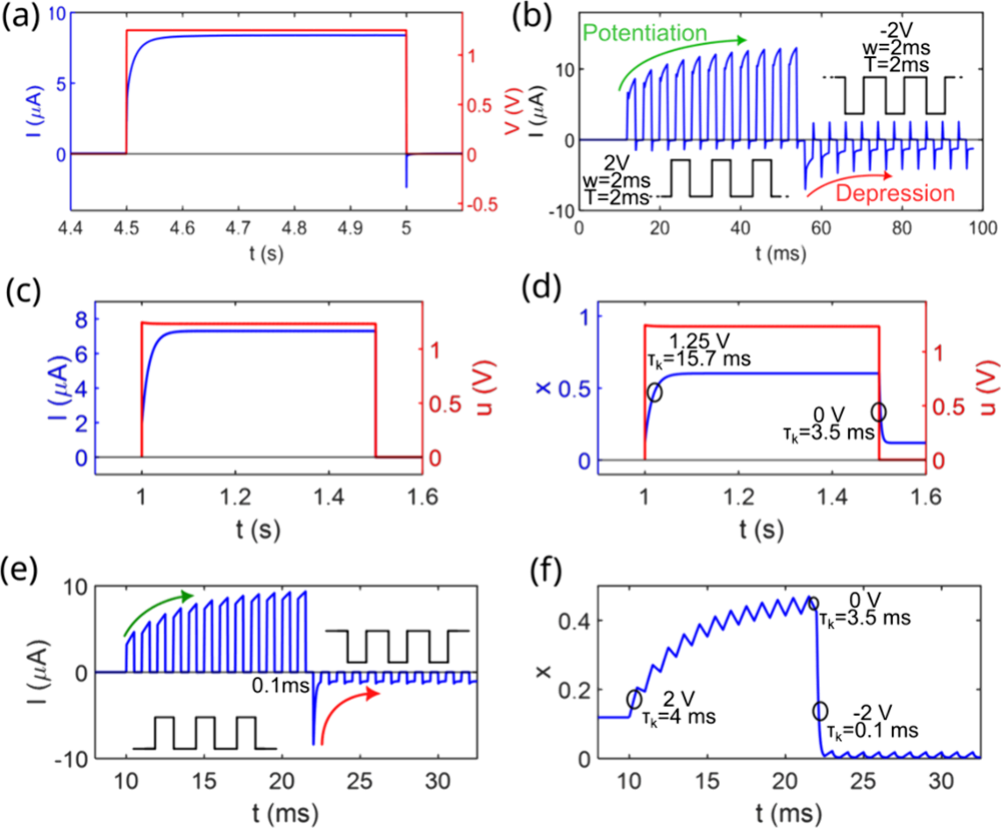
Experimental system current responses when excited with different voltage pulse configuration: (a) A single long (500 *ms*, reaching the stationary current) pulse of 1.25 *V* and (b) two consecutive pulse trains, each with a pulse width of 2 *ms*, a 2*ms* separation between them and amplitudes of +2 *V* and – 2 *V*, respectively. Simulation of neuromorphic properties of the system: (c) EPSC after a 500 *ms*, 1.25 *V* pulse (note that the internal voltage of the system is shown, excluding the effects of contact resistance), (d) behavior of the variable *x* during this pulse, (e) potentiation-depression dynamics using the same pulse configuration as in (b), and (f) evolution of the variable *x* during this sequence. Additionally, specific values of the relaxation time corresponding to the different applied voltages are included to provide deeper insight into the internal processes.

This internal memory variable of the system will depend on both the width of the pulses applied and the distance between them. To observe this dependency, the system will be subjected to pairs of pulses, each with an amplitude of 1 *V*, while progressively increasing the distance between each pair and the pulse width within each group of pairs. Subsequently, the percentage increase in the system current will be calculated for each pair of pulses at varying distances and widths. This purely neuromorphic phenomenon is known as paired-pulse facilitation (PPF) and is fully illustrated in Figure 7.

**Figure 7:**
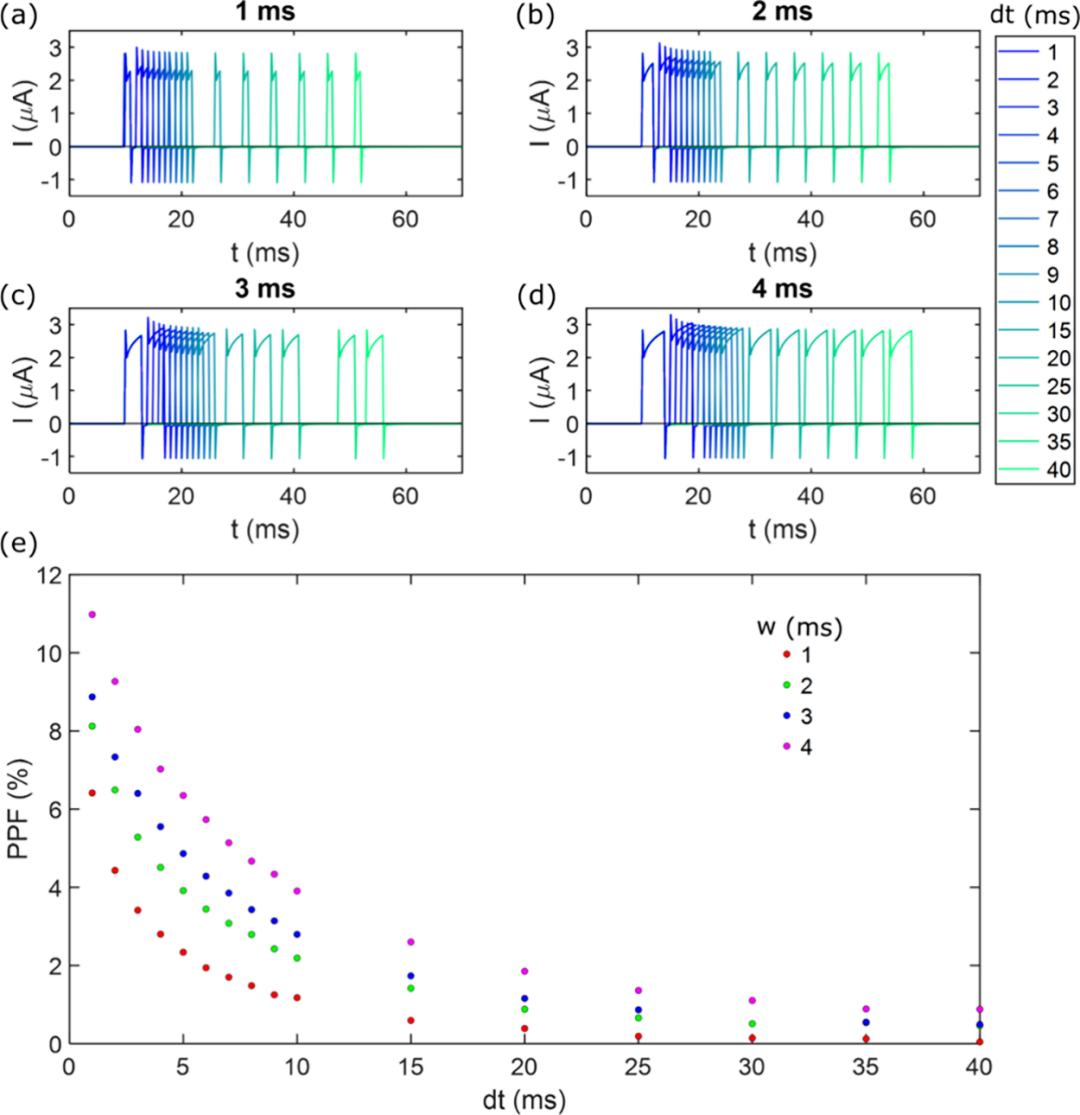
Experimental PPF measurements in our system. (a), (b), (c) and (d) represent the EPSC for different pulse separation, where the darkest blue corresponds to the minimum separation of 1 *ms* and the lightest green corresponds to the maximum separation of 40 *ms*. The pulse widths are 1 *ms*, 2 *ms*, 3 *ms* and 4 *ms*, respectively. (e) Representation of the percentage change in the EPSC for each pair of pulses at varying pulse widths and separations.

In Figure 7, we observe that the broader the pulses and the shorter the separation between them, the greater the increase in consecutive EPSCs. This follows the typical response exhibited by natural synapses.^52^

Building on the model described in eqs 2–5 for the steady-state system, and using the system dynamics derived from the relaxation time in eqs 22 and 25, and Figure 5, we simulate these neuromorphic properties and compare them to experimental neuromorphic responses. This comparison will validate the described models and highlight the potential development of neuromorphic applications achievable simply by understanding basic system parameters.

To this end, we begin by subjecting the system to different pulse configurations. As shown in Figure 6, we first apply a long 1.25 *V* pulse, allowing the system current to reach the stationary. Subsequently, a pair of pulse trains with amplitudes of +2 *V* and – 2 *V*, respectively, will be applied. The aim is to theoretically replicate the behavior of the EPSC, including the potentiation and depression exhibited by the system.

The theoretical EPSC and the system potentiation-depression are shown in Figures 6c and 6e, respectively, while Figures 6d and 6f display the behavior of the system internal memory variable *x* and the relaxation time values corresponding to each applied voltage. These graphs demonstrate a satisfactory correlation with the experimental performance of the system. Moreover, the graphs describing the variable *x* provide deeper insights into the internal processes and the mechanisms driving the transitions over the previously described relaxation time.

To replicate the PPF behavior exhibited by the system, the same pulse configuration will be applied theoretically, maintaining the same voltage value, pulse width, and intervals between pulses. This approach yields the EPSC configurations obtained for each pulse width, as shown in Figures 8(a-d), similar to those of Figures 7(a-d). Subsequently, Figure 8e presents the theoretical PPF obtained for the system, which is compared to the experimental PPF shown in Figure 7e.

**Figure 8:**
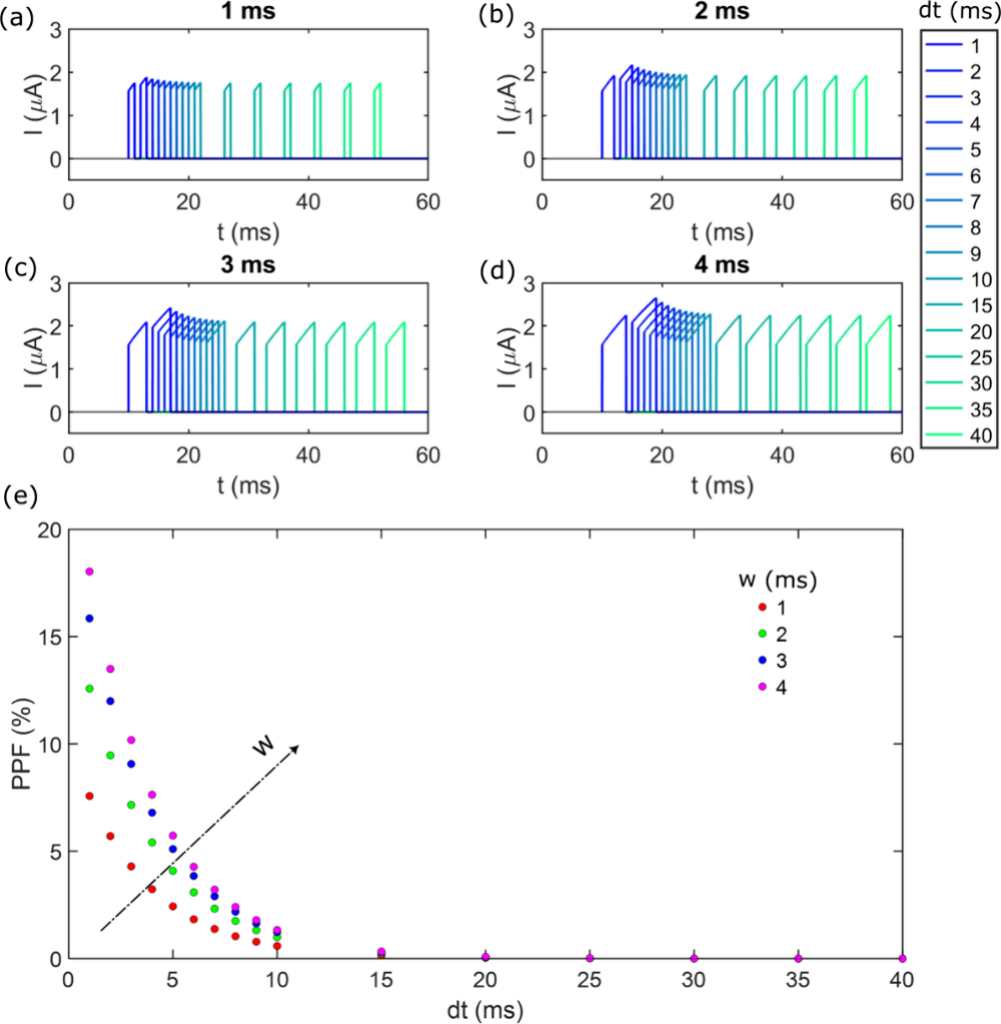
Simulated PPF. (a), (b), (c) and (d) represent the EPSC for different pulse separation, where the darkest blue corresponds to the minimum separation of 1 *ms* and the lightest green corresponds to the maximum separation of 40 *ms*. The pulse widths are 1 *ms*, 2 *ms*, 3 *ms* and 4 *ms*, respectively. (e) Representation of the percentage change in the EPSC for ech pair of pulses at varying pulse widths and separations.

## 4 Discussion

This work describes the model for the electrical response of our memristor device (eqs 2–5), including the chemical-physical origin of the memory state variable *x* (eq 1). We fitted the model parameters to the stationary current–voltage curve shown in Figure 2a, with the simulation in Figure 2c closely matching the experimental response.

Subsequently, we applied the small-signal AC impedance response to the model and represented key parameters such as the model conductances. To verify the correctness of the model (eqs 2–3), we performed IS measurements, confirming that the obtained parameters followed the expected theoretical behavior. We introduce an RC branch to model ionic accumulation at large positive voltages, obtaining parameters *R*_*i*_ and *C*_*i*_. These parameters increase the conductances and introduce noise, which cannot be accounted for by the model in eqs 2 and 3, leading to a longer system response time. In Figure 4f and 4g, we observe a rise in these parameters beyond 2 V, indicating the increasing influence of ionic accumulation on the system dynamics. We note an increase in all experimental conductances of the Figure 4c compared to the expected values from the simulation shown in Figure 2e, slightly higher values of capacitance *C*_*m*_ shown in Figure 4d, and a change in the slope of the relaxation time decay beyond 2 V, shown in Figure 5. Therefore, we can conclude that ahead of this voltage, a combination of the previously well-defined model and an ionic charge accumulation effect at the pore contributes to noise in the model. Anyway, this does not affect the device application or its ability to capture the full dynamics of the system because the range where this ionic effect emerges begins at the end tail of the relaxation time decay.

In the absence of an accurate relaxation time model valid across the full voltage range of *x* (0 to 1), it is difficult to describe the kinetics of the conductance transition and chemical inductor, which are crucial to many memristors.^43^ To address this limitation, we derived a compact model for the relaxation time, based on the system’s internal parameters, including those related to the electrochemical cell solution, nanopores, and membrane. Furthermore, we propose two ways to describe this model. In one approach, all parameters are explicitly detailed in the model (22), while in the other, we combine several parameters to derive a typical RC-like form of the relaxation time, eq 26, using the transverse electrical resistance of the device and the chemical capacitor, which strongly correlates with the internal functioning of memristors.^23,33,35,43^ Although a systematic exploration is beyond the scope of this work, this formulation provides a theoretical basis to investigate how physical parameters such as pore geometry, surface charge, or ionic strength may influence the voltage-dependent relaxation time τ_*k*_.

Once established, this model correlates with the system’s intrinsic parameters, providing a comprehensive understanding of fluidic memristors’ dynamic and static operation. As shown in Figure 5, the model satisfactorily fits the experimental data, with the observed increase in values attributed to the ionic accumulation effect discussed earlier (Figure 4f and 4g). At this point, we could relate the fitting parameters to the known characteristics of the pore to extract values for the remaining pore properties. Thus, for the first time we can extract the kinetic time from an impedance spectroscopy analysis, getting as a result a voltage dependence like those of natural neuron ion channels, leading to a prospective horizon in the integration of fluidic nanopore memristors in neuromorphic architectures.

Finally, we conducted neuromorphic measurements such as EPSC, potentiation-depression and PPF, experimentally and through simulations, to further validate the proposed models in real applications of the device. Then, we observe that the system satisfactorily follows the expected trend in EPSC and in potentiation-depression behavior of those in Figures 6, and regarding changes in pulse separation and width of those for PPF in Figures 7 and 8. It is important to consider, however, that in this comparison, exactly the same magnitude values for the curves are not achieved due to the pore sensitivity to dilation at its tip as a result of carrier passage and passive ionic charge accumulation. In other words, because these experimental measurements are performed sequentially, the pore gradually dilates, also altering the ionic charge distribution within it over time. Thus, this difference in the values of the PPF can be disregarded, and it can be concluded that theoretical results satisfactorily align with the experimental observations.

With these results, we can confirm both predicted models: the one previously studied for the static behavior of the system and its relationship with the relaxation time model obtained in this work. In the extensive research on nanofluidic memristors and their extrapolation to other memristors with similar electrical properties, this represents an excellent starting point for establishing control and correlations between the system internal parameters, modeling, and their application in various fields under dynamic regimes, such as neuromorphic computing.

This model could then be scaled for use with other types of memristors, which, while based on different chemical-physical principles, exhibit similar neuromorphic properties and current–voltage characteristics.^33,34,44^ This makes it an excellent starting point for achieving full kinetic and dynamic control over other memristors. Moreover, it could provide valuable insights into the internal mechanisms of these systems, which are often highly complex and challenging to comprehend and regulate.

## 5 Conclusions

Our study demonstrates the potential of memristors for neuromorphic systems through the analysis of IS spectra. By focusing on asymmetric nanopores, we identified the key characteristic of an inductive low-frequency arc, indicating strong potential for these memristors in synaptic applications. Our models reveal voltage-dependent characteristics, closely matching the experimental IS data, and propose a physically grounded model for nanopore relaxation time, marking a significant advancement in understanding fluidic memristors.

This relaxation time model completes the response model for fluidic memristors and has been validated through its application to neuromorphic responses such as potentiation-depression and PPF. These results align well with experimental observations, reinforcing the accuracy and relevance of the proposed model. This framework offers a foundation for more precise control of memristors’ internal parameters and can be extended to other types of memristors with similar neuromorphic properties, allowing for further exploration of their potential in advanced memory and AI applications.

## Supplementary Material



## Data Availability

The data presented here can be accessed at 10.5281/zenodo.15299324 (Zenodo) under the license CC-BY-4.0 (Creative Commons Attribution-ShareAlike 4.0 International).

## References

[1] Christensen D. V., Dittmann R., Linares-Barranco B., Sebastian A., Le Gallo M., Redaelli A., Slesazeck S., Mikolajick T., Spiga S., Menzel S. (2022). 2022 roadmap
on neuromorphic computing and engineering. Neuromorph.
Comput. Eng..

[2] Ielmini D., Wang Z., Liu Y. (2021). Brain-inspired computing
via memory
device physics. APL Mater..

[3] Zidan M. A., Strachan J. P., Lu W. D. (2018). The future
of electronics based on
memristive systems. Nat. Electron..

[4] Saleh S., Koldehofe B. (2022). On Memristors
for Enabling Energy Efficient and Enhanced
Cognitive Network Functions. IEEE Access.

[5] Liu S., Wang Y., Fardad M., Varshney P. K. (2018). A Memristor-Based
Optimization Framework for Artificial Intelligence Applications. IEEE Circ. Syst. Mag..

[6] Sebastian A., Le Gallo M., Khaddam-Aljameh R., Eleftheriou E. (2020). Memory devices
and applications for in-memory computing. Nat.
Nanotechnol..

[7] Miranda E., Suñé J. (2020). Memristors for Neuromorphic Circuits
and Artificial
Intelligence Applications. Materials.

[8] Xiao X., Hu J., Tang S., Yan K., Gao B., Chen H., Zou D. (2020). Recent Advances in
Halide Perovskite Memristors: Materials, Structures,
Mechanisms, and Applications. Adv. Mater. Technol..

[9] Robin P., Kavokine N., Bocquet L. (2021). Modeling of
emergent memory and voltage
spiking in ionic transport through angstrom-scale slits. Science.

[10] Wu F., Cao P., Peng Z., Ke S., Cheng G., Cao G., Jiang B., Ye C. (2022). Memristor
Based on TiOx/Al2O3 Bilayer
as Flexible Artificial Synapse for Neuromorphic Electronics. IEEE Trans. Electron Devices.

[11] Poddar S., Zhang Y., Gu L., Zhang D., Zhang Q., Yan S., Kam M., Zhang S., Song Z., Hu W. (2021). Down-Scalable
and Ultra-fast Memristors with Ultra-high Density Three-Dimensional
Arrays of Perovskite Quantum Wires. Nano Lett..

[12] Park H.-L., Kim M.-H., Kim H., Lee S.-H. (2021). Self-Selective Organic
Memristor by Engineered Conductive Nanofilament Diffusion for Realization
of Practical Neuromorphic System. Adv. Electron.
Mater..

[13] Zhang W., Gao H., Deng C., Lv T., Hu S., Wu H., Xue S., Tao Y., Deng L., Xiong W. (2021). An ultrathin memristor
based on a two-dimensional WS2/MoS2 heterojunction. Nanoscale.

[14] Cordero A., Torregrosa J. R., Bisquert J. (2025). Bifurcation and oscillations in fluidic
nanopores: A model neuron for liquid neuromorphic networks. Phys. Rev. Research.

[15] Hodgkin A. L., Huxley A. F. (1952). A quantitative description
of membrane current and
its application to conduction and excitation in nerve. J. Physiol..

[16] Menzel S., Tappertzhofen S., Waser R., Valov I. (2013). Switching kinetics
of electrochemical metallization memory cells. Phys. Chem. Chem. Phys..

[17] Yang J. J., Strukov D. B., Stewart D. R. (2013). Memristive
devices for computing. Nat. Nanotechnol..

[18] Lee J. S., Lee S., Noh T. W. (2015). Resistive switching phenomena: A review of statistical
physics approaches. Appl. Phys. Rev..

[19] Cüppers F., Menzel S., Bengel C., Hardtdegen A., von Witzleben M., Böttger U., Waser R., Hoffmann-Eifert S. (2019). Exploiting
the switching dynamics of HfO2-based ReRAM devices for reliable analog
memristive behavior. APL Mater..

[20] Dutta M., Brivio S., Spiga S. (2024). Unraveling
the Roles of Switching
and Relaxation Times in Volatile Electrochemical Memristors to Mimic
Neuromorphic Dynamical Features. Adv. Electron.
Mater..

[21] Kamsma T. M., Boon W. Q., ter Rele T., Spitoni C., van Roij R. (2023). Iontronic
Neuromorphic Signaling with Conical Microfluidic Memristors. Phys. Rev. Lett..

[22] Kamsma T. M., Kim J., Kim K., Boon W. Q., Spitoni C., Park J., van Roij R. (2024). Brain-inspired
computing with fluidic iontronic nanochannels. Proc. Natl. Acad. Sci. U.S.A..

[23] Bisquert J. (2024). Hysteresis,
Rectification, and Relaxation Times of Nanofluidic Pores for Neuromorphic
Circuit Applications. Adv. Physics Res..

[24] Ling Y., Yu L., Guo Z., Bian F., Wang Y., Wang X., Hou Y., Hou X. (2024). Single-Pore Nanofluidic Logic Memristor with Reconfigurable
Synaptic Functions and Designable Combinations. J. Am. Chem. Soc..

[25] Emmerich T., Teng Y., Ronceray N., Lopriore E., Chiesa R., Chernev A., Artemov V., Di Ventra M., Kis A., Radenovic A. (2024). Nanofluidic
logic with mechano–ionic memristive
switches. Nat. Electron..

[26] Xiong T., Li W., Yu P., Mao L. (2023). Fluidic memristor: Bringing chemistry
to neuromorphic devices. Innovation.

[27] Ramirez P., Gómez V., Cervera J., Mafe S., Bisquert J. (2023). Synaptical
Tunability of Multipore Nanofluidic Memristors. J. Phys. Chem. Lett..

[28] Ramirez P., Portillo S., Cervera J., Nasir S., Ali M., Ensinger W., Mafe S. (2024). Neuromorphic
responses of nanofluidic
memristors in symmetric and asymmetric ionic solutions. J. Chem. Phys..

[29] Portillo S., Manzanares J. A., Ramirez P., Bisquert J., Mafe S., Cervera J. (2024). pH-Dependent
Effects in Nanofluidic Memristors. J. Phys.
Chem. Lett..

[30] Law C. S., Wang J., Nielsch K., Abell A. D., Bisquert J., Santos A. (2025). Recent advances in fluidic neuromorphic
computing. Appl. Phys. Rev..

[31] Bou A., Bisquert J. (2021). Impedance Spectroscopy
Dynamics of Biological Neural
Elements: From Memristors to Neurons and Synapses. J. Phys. Chem. B.

[32] Ramirez P., Cervera J., Nasir S., Ali M., Ensinger W., Mafe S. (2024). Electrochemical impedance spectroscopy
of membranes with nanofluidic
conical pores. J. Colloid Interface Sci..

[33] Gonzales C., Bou A., Guerrero A., Bisquert J. (2024). Capacitive and Inductive Characteristics
of Volatile Perovskite Resistive Switching Devices with Analog Memory. J. Phys. Chem. Lett..

[34] Bou A., Gonzales C., Boix P. P., Vaynzof Y., Guerrero A., Bisquert J. (2025). Kinetics of Volatile
and Nonvolatile Halide Perovskite
Devices: The Conductance-Activated Quasi-Linear Memristor (CALM) Model. J. Phys. Chem. Lett..

[35] Bisquert J., Roldán J. B., Miranda E. (2024). Hysteresis in memristors produces
conduction inductance and conduction capacitance effects. Phys. Chem. Chem. Phys..

[36] Cervera J., Ramirez P., Mafe S., Stroeve P. (2011). Asymmetric nanopore
rectification for ion pumping, electrical power generation, and information
processing applications. Electrochim. Acta.

[37] Cervera J., Schiedt B., Ramírez P. (2005). A Poisson/Nernst-Planck
model for ionic transport through synthetic conical nanopores. Europhys. Lett..

[38] Cervera J., Schiedt B., Neumann R., Mafé S., Ramírez P. (2006). Ionic conduction, rectification, and selectivity in
single conical nanopores. J. Chem. Phys..

[39] Bisquert J., Sánchez-Mateu M., Bou A., Suwen Law C., Santos A. (2024). Synaptic Response of Fluidic Nanopores:
The Connection
of Potentiation with Hysteresis. ChemPhysChem.

[40] Bisquert J. (2023). Current-controlled
memristors: Resistive switching systems with negative capacitance
and inverted hysteresis. Phys. Rev. Appl..

[41] Berruet M., Pérez-Martínez J. C., Romero B., Gonzales C., Al-Mayouf A. M., Guerrero A., Bisquert J. (2022). Physical Model for
the Current–Voltage Hysteresis and Impedance of Halide Perovskite
Memristors. ACS Energy Lett..

[42] Jamnik J., Maier J. (2001). Generalised equivalent
circuits for mass and charge transport: chemical
capacitance and its implications. Phys. Chem.
Chem. Phys..

[43] Bisquert J., Guerrero A. (2022). Chemical Inductor. J. Am. Chem.
Soc..

[44] Bisquert J. (2024). Inductive
and Capacitive Hysteresis of Current-Voltage Curves: Unified Structural
Dynamics in Solar Energy Devices, Memristors, Ionic Transistors, and
Bioelectronics. PRX Energy.

[45] Kim S.-Y., Zhang H., Rivera-Sierra G., Fenollosa R., Rubio-Magnieto J., Bisquert J. (2025). Introduction to neuromorphic
functions
of memristors: The inductive nature of synapse potentiation. J. Appl. Phys..

[46] Cervera J., Portillo S., Ramirez P., Mafe S. (2024). Modeling of memory
effects in nanofluidic diodes. Phys. Fluids.

[47] Wilson, H. R. Spikes, Decisions, and Actions: The Dynamical Foundations of Neuroscience; Oxford University Press: 1999.

[48] Hopper A. J., Beswick-Jones H., Brown A. M. (2022). A color-coded graphical
guide to
the Hodgkin and Huxley papers. Adv. Physiol.
Educ..

[49] Bisquert J. (2023). Electrical
Charge Coupling Dominates the Hysteresis Effect of Halide Perovskite
Devices. J. Phys. Chem. Lett..

[50] Shi D., Wang W., Liang Y., Duan L., Du G., Xie Y. (2023). Ultralow Energy
Consumption Angstrom-Fluidic Memristor. Nano
Lett..

[51] Zhang P., Xia M., Zhuge F., Zhou Y., Wang Z., Dong B., Fu Y., Yang K., Li Y., He Y. (2019). Nanochannel-Based
Transport in an Interfacial Memristor Can Emulate the Analog Weight
Modulation of Synapses. Nano Lett..

[52] Zucker R. S., Regehr W. G. (2002). Short-Term Synaptic Plasticity. Annu. Rev. Physiol..

