# Supplementary material for: Relaxation Time of Multipore Nanofluidic Memristors for Neuromorphic Applications
Source: J Am Chem Soc. 2025 May 11;147(20):17529–38. doi: 10.1021/jacs.5c04903 (PMC12100719; doi:10.1021/jacs.5c04903)
Supplement: Supplementary file 1 [file ja5c04903_si_001.pdf]

Supporting Information

Relaxation Time of Multipore Nanofluidic Memristors for Neuromorphic Applications

Gonzalo Rivera-Sierra,[1] Patricio Ramirez,[2] Juan Bisquert,[1] Agustín Bou*[3]

[1]Instituto de Tecnología Química (Universitat Politècnica de València-Consejo Superior de Investigaciones Científicas), Av. dels Tarongers, 46022, València, Spain.

[2]Dept. de Física Aplicada, Universitat Politècnica de València, E-46022 València, Spain

[3]Leibniz-Institute for Solid State and Materials Research Dresden, Helmholtzstraße 20, 01069 Dresden, Germany

Email: a.bou.catala@ifw-dresden.de

Experimental details

The multipore membranes were obtained using the track-etching technique.[1] In short, 12.5 μm thick polyimide foils (Kapton50 HN, DuPont) were irradiated by swift heavy ions at the linear accelerator UNILAC (GSI, Darmstadt). The resulting latent tracks were converted into approximately conical nanopores by asymmetric etching with concentrated NaOH. The dimensions of the nanopores were estimated by fitting the experimental current–voltage (I–V) curves using a Poisson–Nernst–Planck (PNP) model, following the approach established in previous studies that correlate I–V characteristics with pore geometry measured by SEM.[2, 3]

The pore geometry is described by the following exponential equation:

$$a(x) = \frac{a_R - a_L\, exp\left[-(d/h)^n\right] - (a_R - a_L)\, exp\left[-(x/d)^n (d/h)^n\right]}{1 - exp\left[-(d/h)^n\right]} \quad (n > 0) \tag{S1}$$

where x is the coordinate along the pore axis, d is the pore length, a_L and a_R are the radii of the tip and base, respectively, and n and d/h are parameters controlling the curvature of the pore profile. In the case of pores with a bullet-shaped tip, $n \approx 1$, while d/h grows as the pore separates from the conical shape (the limit $d/h \to 0$ corresponds to exactly conical pores).

Electrical conductance measurements combined with previous SEM studies reveal pore diameters of the orders of 200 nm (cone base) and 20 nm (cone tip). In our case, a good fit was obtained using $d = 12.5$ μm, $a_L = 15$ nm, $a_R = 280$ nm, $d/h = 5$, and a surface charge density $s = 0.8q$, where q is the elementary charge. These values are consistent with previous nanopore fabrication protocols using the same materials and etching conditions.

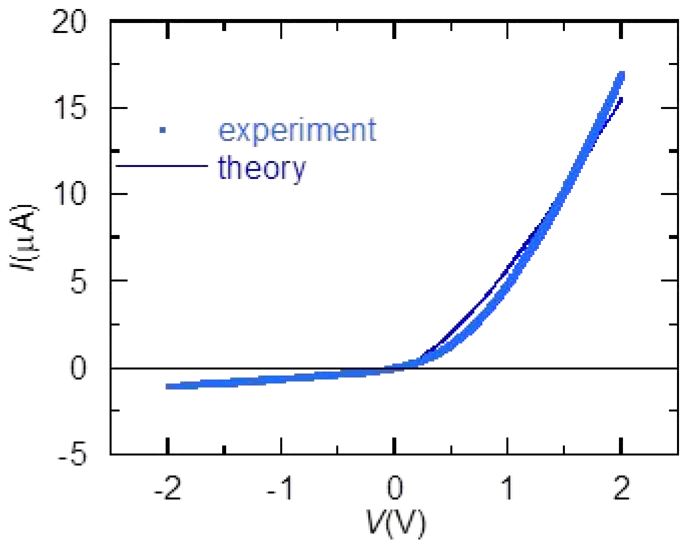

Figure S1. Current voltage curves of a nanoporous membrane containing approximately 300 pores. The experimental curves (points) were obtained with 100 mM KCl solutions at neutral pH. The theoretical curves (continuous lines) were calculated assuming that the membrane sample contained 300 nanopores with a surface charge $s = 0.8q$, and using $d = 12.5$ μm, $a_L = 15$ nm, $a_R = 280$ nm and $d/h = 5$ in the theoretical model of references [2, 3]

The etching process results also in carboxylate residues on the pore surface that provide negative fixed pore charge densities at neutral pH, in the range between -0.1 and $-1.0\ q/nm^2$ (q is the elementary charge). The charged membranes produced by this process show ionic selectivity, promoting the passage of counter-ions (ions of opposite charge to those bound to the pores) but preventing the permeation of co-ions (ions of the same charge as that of the pores).

For the experiments, the membrane sample was placed separating the two half chambers of an electrodialysis cell, as shown in Fig. 1. The exposed membrane area was $1\ cm^2$. The cell was placed within a double-layer magnetic shield (Amuneal, Philadelphia) which was mounted on an anti-vibration table (Technical Manufacturing Corporation, Peabody, Massachusetts). Each cell chamber was filled with $100\ mM$ KCl aqueous solution at neutral pH. All experiments were conducted with a BioLogic SP-200 potentiostat (Seyssinet-Pariset, France). Currents and voltages were introduced in the solutions by two Ag|AgCl electrodes with $2\ M$ KCl salt solution bridges. Steady-state current-voltage (I-V) curves were recorded with $2\ V$ amplitude, triangular voltage signals with a sweep rate of $200\ mV/s$. I-V curves showing memristive hysteretic loops were measured with sinusoidal, $2\ V$ amplitude voltage signals of frequencies in the range $1 - 100\ Hz$. In our measurements, $I > 0$ and $V > 0$ corresponded to electric currents entering the pore tip. IS spectra, as well as current and voltage pulse sequences in synaptic measurements were measured also using the same sign convention. To obtain the IS spectra, $10\ mV$ amplitude sinusoidal voltage waves with frequencies in the range between

$100\ mHz$ and $200\ kHz$ were superposed to the fixed DC applied voltage.

The multipore membranes obtained show stable rectifying steady state I-V curves at frequencies lower than $100\ mHz$, with a high conducting state for $V > 0$ and a low conducting state for $V < 0$. At frequencies in the range $1 - 100\ Hz$ the membranes show memristic-like pinched I-V curves with an inductive loop at $V > 0$ and a capacitive loop for $V < 0$.

(1) Ramirez, P.; Gómez, V.; Cervera, J.; Mafe, S.; Bisquert, J. Synaptical Tunability of Multipore Nanofluidic Memristors. *J. Phys. Chem. Lett.* **2023**, *14* (49), 10930-10934. DOI: 10.1021/acs.jpclett.3c02796.

(2) Cervera, J.; Schiedt, B.; Ramírez, P. A Poisson/Nernst-Planck model for ionic transport through synthetic conical nanopores. *Europhys. Lett.* **2005**, *71* (1), 35. DOI: 10.1209/epl/i2005-10054-x.

(3) Cervera, J.; Schiedt, B.; Neumann, R.; Mafé, S.; Ramírez, P. Ionic conduction, rectification, and selectivity in single conical nanopores. *J. Chem. Phys.* **2006**, *124* (10), 104706. DOI: 10.1063/1.2179797 (acccessed 3/19/2025).
